# Decision-making regarding anticoagulant therapy in the last phase of life: In-depth interview study with healthcare professionals (the OPTIMA trial)

**DOI:** 10.1177/02692163251394875

**Published:** 2025-11-26

**Authors:** Marte A. M. van Hylckama Vlieg, Karlijn J. I. de Beukelaar, Romy H. L. Spreeuw, Marieke J. H. A. Kruip, P. Hugo M. van der Kuy, Agnes van der Heide, Carin C. D. van der Rijt, Eric C. T. Geijteman

**Affiliations:** 1 Department of Medical Oncology, Erasmus MC Cancer Institute, Rotterdam, The Netherlands; 2 Department of Hematology, Erasmus MC, University Medical Center Rotterdam, Rotterdam, The Netherlands; 3 Department of Quality and Patient Care, Erasmus MC, University Medical Center Rotterdam, Rotterdam, The Netherlands; 4 Department of Hospital Pharmacy, Erasmus MC, University Medical Center Rotterdam, Rotterdam, The Netherlands; 5 Department of Public Health, Erasmus MC, University Medical Center Rotterdam, Rotterdam, The Netherlands

**Keywords:** anticoagulants, thromboembolism, hemorrhage, clinical decision-making, palliative care, qualitative research

## Abstract

**Background:**

Many patients in the last phase of life use anticoagulants. There is little evidence on the benefits and risks of (dis)continuation of anticoagulant therapy in this phase, leaving healthcare professionals with a complex clinical dilemma.

**Aim:**

To gain an in-depth understanding of the experiences, perspectives, and preferences of healthcare professionals regarding decision-making about anticoagulant therapy in the last phase of life.

**Design:**

Semi-structured in-depth interviews were conducted and analyzed using a thematic content analysis.

**Setting/participants:**

This study was performed in the Dutch Federation of Anticoagulation Clinics, two hospitals, two hospices, and two palliative home care groups in the Netherlands. Thirty-two healthcare professionals regularly involved in care for patients in the last phase of life were interviewed.

**Results:**

Healthcare professionals experienced decision-making regarding anticoagulant therapy in the last phase of life as difficult and multifactorial, due to a lack of knowledge on the incidence of events after (dis)continuing therapy, the uncertain risk trade-off between events with major consequences, the variety of indications for therapy, the variety of comorbidities and patient characteristics, and prognostic uncertainty. As a result, healthcare professionals act defensively when considering discontinuation, due to concerns about being held accountable for potential thrombotic events. Uncertainty in decision-making results in reactive decision-making in response to triggers such as active bleeding, low performance score, recognition of the dying phase, and patient’s preference.

**Conclusions:**

This study highlighted the complexity of decision-making regarding anticoagulant therapy in the last phase of life. This results, in practice, in reactive instead of proactive decision-making.

**What is already known about the topic?**

- Many patients in the last phase of life use anticoagulants
- The appropriateness of anticoagulant therapy requires careful balancing of the treatment and prevention of thromboembolic events against the risks of minor and major bleeding
- There is little evidence on the benefits and risks of (dis)continuation of anticoagulant therapy in the last phase of life, leaving healthcare professionals with a complex clinical dilemma

**What this paper adds?**

- Healthcare professionals experience decision-making regarding anticoagulant therapy in the last phase of life as difficult and multifactorial
- This paper identifies several key factors influencing healthcare professionals’ decision-making regarding anticoagulant therapy in the last phase of life. These factors include clinical reasoning, conscious consideration of (dis)continuation following impactful experiences, prognostic uncertainty, defensive decision-making driven by concerns about being held accountable for potential thrombotic events, and the patient’s preference
- Uncertainty in decision-making results in reactive, rather than proactive, decision-making in response to triggers

**Implications for practice, theory, or policy**

- It is essential to understand how patients perceive anticoagulant therapy in the last phase of life and how they wish to be involved in shared decision-making
- Knowledge of the incidence of events after (dis)continuing anticoagulant therapy is needed for decision-making. Future research should focus on studies stratified by indication for anticoagulant therapy and relevant comorbidities

## Introduction

Anticoagulants are prescribed to prevent or treat venous and arterial thromboembolism, and thereby aim to improve patients’ prognosis and quality of life. Anticoagulants are classified into three groups: vitamin K antagonists, low-molecular-weight heparins, and direct oral anticoagulants. Many patients in the last phase of life use anticoagulants.^1–7^ An observational study showed that 33.5% of Dutch patients with a life expectancy of less than 3 months, used anticoagulants, and almost half of these patients still used anticoagulants on the day of death.^3^ The most common indication for anticoagulant therapy in the last phase of life is the prevention of thromboembolic events due to atrial fibrillation, accounting for 42%–53% of patients receiving anticoagulant therapy in this phase.^2,8^ Other common reasons include the prevention and treatment of venous thrombosis, the prevention of thromboembolic events after heart valve replacement, and some arterial diseases.^2^

Anticoagulants are often continued until death, independent of the indication at start and treatment goal.^8^ In the last phase of life, when curing and prolonging life is no longer possible, pharmacological treatment should focus on symptom management and optimizing patients’ quality of life.^9^ In line with this shift in goals, consensus has been reached that certain medications, such as lipid-lowering medication, are inappropriate for patients with a limited life expectancy.^10–12^ In contrast, the appropriateness of anticoagulant therapy is a more complex topic, as it involves balancing the treatment and prevention of thromboembolic events with the risk of minor and major bleedings.^13^

There is little evidence on the benefits and risks of (dis)continuation of anticoagulant therapy in the last phase of life.^13–15^ Although research is gradually emerging for patients with advanced cancer,^16–20^ large-scale interventional studies are still lacking. Research is very limited for patients with life-limiting diseases other than advanced cancer. As a result, evidence-based guidance regarding anticoagulant therapy in the last phase of life is lacking. For certain indications, such as atrial fibrillation, clinical guidelines suggest lifelong anticoagulant therapy. However, they offer limited guidance on when therapy should be reconsidered or discontinued in patients in the last phase of life.^21,22^ This leaves healthcare professionals with complex clinical dilemmas.^23,24^ Previous studies on physicians’ perspectives regarding these dilemmas are mostly related to patients with advanced cancer.^13,25–30^ The only study that was related to a more diverse group of patients in the last phase of life was conducted by Huisman et al.^13^ which involved a secondary analysis of interviews with physicians, and therefore did not explore this dilemma in-depth.

Therefore, this study aimed to investigate the experiences, perspectives, and preferences of healthcare professionals regarding decision-making about anticoagulant therapy in the last phase of life.

## Methods

### Research question

What are the experiences, perspectives, and preferences of healthcare professionals regarding decision-making about anticoagulant therapy in the last phase of life?

### Design

This multicenter qualitative study is part of the OPTIMA (Toward OPTIMizing Anticoagulant therapy in the last phase of life) project. The OPTIMA project is a mixed-methods study to gain an in-depth understanding of the experiences and perspectives of patients, their relatives, and healthcare professionals regarding anticoagulant therapy in the last phase of life. In this qualitative study, we conducted in-depth interviews with healthcare professionals regularly involved in care for patients in the last phase of life, and analyzed the data using thematic content analysis. Reporting was guided by the Consolidated Criteria for Reporting Qualitative Research (COREQ).^31^

### Setting

This study was performed in the Dutch Federation of Anticoagulation Clinics (FNT), two hospitals, two hospices, and two palliative home care groups in the Netherlands. In a palliative home care group, general practitioners and district nurses regularly meet to identify patients who need palliative care and discuss how they can provide them the best palliative care.

### Population

Healthcare professionals who were regularly involved in care for patients in the last phase of life and had experience in prescribing and managing anticoagulant therapy in this phase were eligible for inclusion. The last phase of life was defined as the phase in which the attending healthcare professional “would not be surprised if the patient would die within one year” (“Surprise question”).^32^ Anticoagulant therapy was defined as the use of low molecular weight heparin, vitamin K antagonists, or direct oral anticoagulants. Healthcare professionals included physicians, nurses, and nurse practitioners. Healthcare professionals who were unable to participate in an interview conducted in Dutch were not eligible for inclusion.

### Sampling and recruitment

Selection of participating healthcare centers and hospital departments was based on the likelihood that they employed healthcare professionals who were regularly involved in prescribing and managing anticoagulant therapy for patients in the last phase of life. Designated contact persons within these centers were approached to identify which healthcare professionals met the inclusion criteria and, if so, to inform them about the interview study and ask them if they could be approached by the investigators. Healthcare professionals who were willing to participate in the study received written information about the interview from the investigators and, upon providing informed consent, were interviewed. Healthcare professionals affiliated with the Dutch Federation of Anticoagulation Clinics (FNT) were also approached for inclusion. Given the size and organizational structure of the FNT, sampling and recruitment were conducted differently: during study presentations for FNT healthcare professionals, the eligibility criteria were outlined, and healthcare professionals were invited to self-identify as eligible and willing to participate. We used a purposive sampling strategy to obtain a diverse study population regarding age, gender, and profession.

### Data collection

Semi-structured in-depth interviews were conducted face-to-face at the workplace of healthcare professionals from November 2023 to January 2025 by three researchers (M.H., medical doctor; K.B. and R.S, medical interns). A topic guide was used to structure the interviews. The topic guide contained open-ended questions on the following main topics on anticoagulant therapy in the last phase of life: current practice of (dis)continuing, considerations and preferences, trade-offs between harmful and beneficial treatment outcomes, and evidence needed to make optimal decisions. The topic guide was refined based on new insights gained during the interviews. The final version is included in Supplemental Material 1. Interviews were conducted until the project group agreed that data saturation had been reached.

### Data analysis

All interviews ranged in length from 30 to 60 min and were audio-recorded. All recordings were transcribed verbatim, anonymized, and analyzed using ATLAS.ti software (v9.90; ATLAS.ti Scientific Software Development GmbH, Berlin). Thematic content analysis was performed. The analysis started with (re)reading the transcripts. In the first phase, 10 transcripts were openly coded by one researcher (M.H. or K.B.) and reviewed by a second researcher (K.B. or M.H.), resulting in a preliminary unstructured list of open codes. In the axial coding phase, relations between codes were identified and categories were created by two researchers (M.H. and K.B). These categories were discussed with a third researcher (E.G., medical doctor) until consensus was reached. The remaining interviews were axially coded by two researchers (M.H. and K.B.), and the resulting codes were reviewed by a third researcher (E.G.). Data saturation was reached when no new codes or categories emerged. In the selective coding phase, the identified categories were discussed and summarized in subthemes and main themes. The final codes, categories, and themes were discussed with the project group. Discrepancies were resolved by discussions between all researchers.

### Ethical issues

The Medical Ethics Review Committee of the Erasmus MC concluded after review of the study protocol that the rules laid down in the Medical Research Involving Human Subjects Act (Dutch abbreviation: WMO) did not apply to this study (MEC-2023-0303). All participants provided written informed consent to participate.

## Results

Thirty two healthcare professionals were interviewed, of whom nine were general practitioners, seven medical oncologists, four specialists in elderly care, and 12 other healthcare professionals. There were 21 female and 11 male participants, with a median age of 42 years (interquartile range 37–58). The characteristics of the interviewed healthcare professionals are shown in Table 1. The thematic content analysis resulted in four main themes and 14 subthemes (Table 2). These themes are discussed below.

**Table 1.** Characteristics interviewed healthcare professionals (*n* = 32).

Participant characteristics	Number of participants, n (%)
Gender	
Female	21 (65.6)
Male	11 (34.4)
Age (median, IQR)	42 (37–58)
Years of professional experience^a^	
<10	15 (46.9)
10–19	8 (25.0)
⩾20	9 (28.1)
Healthcare center	
Hospital	13 (40.6)
PaTz^b^	8 (25.0)
Hospice	6 (18.8)
Thrombosis service	5 (15.6)
Function	
General practitioner	9 (28.1)
Medical oncologist	7 (21.8)
Specialist in elderly care	4 (12.5)
Hospice nurse	2 (6.3)
Nurse practitioner	2 (6.3)
Cardiologist	2 (6.3)
Neurologist	2 (6.3)
General internist	1 (3.1)
General physician	1 (3.1)
Physician assistant	1 (3.1)
Hematologist	1 (3.1)

a Years of professional experience in the current function.

b PaTz: palliative home care group.

**Table 2.** Overview of the themes on decision-making regarding anticoagulant therapy in the last phase of life.

Main themes	Subthemes
1. Perspectives on advantages and disadvantages regarding anticoagulant therapy	1.1 Last months: focus of anticoagulant therapy is improving patients’ quality of life instead of life prolongation
	1.2 Last weeks: the occurrence of a thrombotic event following the discontinuation of anticoagulant therapy may not necessarily be detrimental
2. Reference moments for evaluation of anticoagulant therapy	2.1 Prognostic uncertainty
	2.2 Life expectancy of less than 2 weeks is a clear moment for evaluating and discontinuing anticoagulant therapy
	2.3 A less limited life expectancy increases prognostic uncertainty, providing no clear moment for evaluating anticoagulant therapy
3. Decision-making regarding anticoagulant therapy	3.1 Multifactorial difficulties
	3.2 Clinical reasoning
	3.3 More conscious consideration of (dis)continuing after specific experiences
	3.4 Defensive decision-making in relationship to discontinuation
	3.5 Reactive decision-making based on triggers
	3.6 Patient’s preference
4. Solutions and recommendations	4.1 Guideline is needed, however the complexity of this multifactorial dilemma complicates the creation of a comprehensive guideline
	4.2 Guideline helpful, especially in the shared decision-making process with the patient
	4.3. Future research should focus on discontinuation trials stratified by indication for the anticoagulant

### Perspectives on the advantages and disadvantages regarding anticoagulant therapy

Most healthcare professionals indicated that the general aim of anticoagulant therapy in the last phase of life is not prolonging patients’ life but improving patients’ quality of life. Many healthcare professionals considered the occurrence of a major thrombotic event in the last months of life, for example, a large ischemic stroke resulting in half-sided paralysis, a significant factor in diminishing patients’ quality of life. Therefore, they often continue anticoagulant therapy in these final months.

When a patient is still in good condition, the occurrence of a thrombotic event can be highly disruptive. If I expect them to live another 3 to 6 months, but they suddenly suffer an ischemic stroke and end up in a nursing home with aphasia, that is clearly undesirable. . . Since taking apixaban twice daily is manageable, I choose to continue anticoagulation in such cases. (Participant 26, Medical oncologist)

On the other hand, several healthcare professionals indicated that the occurrence of a thrombotic event following the discontinuation of anticoagulant therapy may not necessarily be detrimental when a patient enters the dying phase. A large thrombotic event in the dying phase resulting in hastening the dying process, may even be a relief of suffering, especially in cases where the dying process proceeds slowly and with significant distress. However, other healthcare professionals indicated that they prefer to avoid large thrombotic events in the dying phase, mainly because of concerns expressed by patients and relatives. These healthcare professionals indicated that their patients considered concerns about bleeding incidents to be less important.

If a patient is in the dying phase and bedridden, I am generally not very concerned about the possibility of an ischemic stroke. Should such an event occur, it may accelerate the dying process. If the patient experiences significant distress, there are medications such as morphine or midazolam available to provide effective symptomatic relief. (Participant 26, Medical oncologist)

In my experience, families and patients often consider the risk of a bleeding far less important than the likelihood of ending up with a hemiparesis in bed due to an ischemic stroke. (Participant 4, General internist)

### Reference moments for evaluation of anticoagulant therapy

Patients’ limited life expectancy is considered an important reference moment for evaluating anticoagulant therapy. However, many healthcare professionals consider it difficult to predict patient’s life expectancy, especially for patients with gradually deteriorating chronic diseases. Due to prognostic uncertainty, healthcare professionals are often reluctant to make decisions regarding anticoagulant therapy. For participants working in anticoagulation clinics, unexplained International Normalized Ratio (INR) fluctuations, sudden INR increase, or an INR difficult to control are signs that the patient is in the last weeks of life.

Well, decision-making regarding anticoagulant therapy starts with the question ‘Can you predict patient’s life expectancy properly?’ I wish I could. (Participant 29, Cardiologist)

Patients’ remaining life expectancy can be better predicted when death is imminent. Estimating life expectancy was thus found to be easier in the hospice setting. Almost all healthcare professionals stated that a life expectancy of less than 2 weeks clearly suggests that discontinuation of anticoagulant therapy should be considered. However, a small number preferred to continue anticoagulant therapy in the dying phase.

Most healthcare professionals experienced greater prognostic uncertainty when a patient’s life expectancy is less limited. This prognostic uncertainty made healthcare professionals less likely to discontinue therapy, as a longer life expectancy increases the likelihood of experiencing a thrombotic event. However, some healthcare professionals indicated that the onset of the terminal phase (less than 3 months life expectancy), if properly estimated, can be a clear reference point to evaluate anticoagulant therapy.

When patients are in their final two weeks of life, I generally discontinue anticoagulant therapy at that point. (Participant 22, Specialist in elderly care)

The likelihood of developing a thrombotic event resulting from your decision to discontinue anticoagulant therapy is inherently limited by the patient’s remaining life expectancy. The shorter the expected survival time, the lower the probability that such an event will occur. (Participant 12, General Practitioner)

### Decision-making regarding anticoagulant therapy

#### Multifactorial difficulties

Despite acknowledging life expectancy as a more or less clear reference point to evaluate anticoagulant therapy, most healthcare professionals found decision-making in the last phase of life difficult and multifactorial. This was mostly experienced by general practitioners. Frequently cited difficulties were a lack of knowledge on the incidence of events after (dis)continuing anticoagulant therapy, the uncertain risk trade-off between different events with major consequences (bleeding vs thrombotic event), prognostic uncertainty, the impact of the event (minor bleeding vs major bleeding, and minor asymptomatic deep vein thrombosis vs major pulmonary embolism), the variety of indications for anticoagulant therapy, and the variety of relevant comorbidities and patient characteristics. In contrast, a small number of healthcare professionals indicated that they did not find decision-making in the last phase of life difficult, as they generally continue anticoagulant therapy.

Choosing to continue or discontinue anticoagulant therapy is not inherently right or wrong. There is no clear consensus on what constitutes the wisest course of action. At this moment, it seems we simply lack definitive knowledge, which is why clinicians approach decision-making regarding anticoagulant therapy in different ways. (Participant 30, Neurologist)

When it comes to patients in the palliative phase who are on anticoagulants, I often feel uncertain. I simply don’t know what the best course of action is, and I frequently find myself questioning whether I’m making the right decisions regarding anticoagulant therapy. (Participant 17, General Practitioner)

#### Healthcare professionals’ clinical reasoning and experiences

Most healthcare professionals stated that they primarily rely on clinical reasoning and intuition, based on previous experiences, when making decisions about anticoagulant therapy in the last phase of life. They make a more conscious and thoughtful decision to (dis)continue therapy after experiencing an impactful event associated with such a decision. This was mentioned equally in both directions: experiencing a bleeding event while continuing anticoagulant therapy versus experiencing a thrombotic event while discontinuing. Some healthcare professionals stated that they believed these experiences may play a disproportionate role in decision-making, because it is frequently grounded in a single experienced case. Furthermore, the more experienced healthcare professionals stated that they felt more confident in decision-making, compared to the younger healthcare professionals. However, many healthcare professionals indicated that, in addition to their clinical reasoning, they would still like these numbers on the incidence of events and guidelines to be available.

My decisions regarding anticoagulant therapy are guided by clinical reasoning and experience. Since I haven’t frequently encountered thrombotic events after discontinuing anticoagulant therapy, I feel more confident in choosing to discontinue anticoagulant therapy. However, for less experienced colleagues, this decision can be more challenging, and in such cases, clear and robust evidence is essential to support clinical choices. (Participant 27, Specialist in elderly care)

At one point, I discontinued the anticoagulant therapy in a frail older patient with a high risk of falling. Shortly thereafter, the patient suffered a major ischemic stroke. This experience was deeply discouraging and has made me more hesitant to discontinue or adjust anticoagulant therapy in the future. (Participant 6, General Practitioner)

#### Defensive and reactive decision-making based on triggers

The majority of healthcare professionals stated that they act defensively when it comes to decision-making regarding anticoagulant therapy. By this, they referred to the perception that if therapy is discontinued and a thrombotic event subsequently occurs, the healthcare professional who made the decision is responsible for that outcome. This is due to the healthcare professional’s active decision to discontinue therapy. These healthcare professionals considered that the occurrence of a bleeding caused by the anticoagulant is perceived as a potential risk of the therapy and is therefore more readily accepted. As a result, these healthcare professionals act defensively regarding discontinuation. In contrast, a few healthcare professionals considered a bleeding event worse compared to a thrombotic event, because of the iatrogenic harm.

Physicians may be reluctant to discontinue anticoagulant therapy due to concerns about being held accountable for potential thrombotic events. If a patient were to experience an ischemic stroke after discontinuing therapy, the responsibility is often perceived to lie with the physician, which can be a scary feeling. (Participant 5, General Physician)

I believe this applies to many physicians, not just myself. Once a particular treatment path has been chosen, it can be difficult to deviate from it. There’s often a fear that if anticoagulant therapy is discontinued and the patient subsequently develops a pulmonary embolism, it will be seen as the consequence of the action of the physician. These concerns can strongly influence clinical decision-making. (Participant 30, Neurologist)

Many healthcare professionals mentioned that they act reactively based on triggers when it comes to decision-making. Frequently mentioned triggers to discontinue anticoagulant therapy were an active bleeding, patients’ inability to swallow the anticoagulant, high risk of falling, low performance score, recognition of the dying phase, and patients’ preference.

In the dying phase, I discontinue all oral medications, including anticoagulants, because patients are no longer able to swallow the anticoagulant. In that context, discontinuation is a logical step. (Participant 24, Medical oncologist)

#### Patient’s preference

Perhaps the most important trigger in decision-making is the patient’s preference. Many healthcare professionals indicated that, when feeling uncertain whether to continue or discontinue anticoagulant therapy, they prefer to discuss this issue with the patient. By presenting the advantages and disadvantages they aim to reach a conclusion through shared decision-making led by the patient’s preference. Furthermore, these healthcare professionals indicated that if they anticipate in advance that the patient may not be receptive to discussing this issue, they are less likely to raise this topic with the patient and are inclined to continue the anticoagulant therapy. This is, among others, related to the patient’s coping mechanism. In contrast, other healthcare professionals indicated that they question whether patients are capable of making an informed decision on this issue, since they cannot properly assess the risks and consequences.

The primary consideration in the decision-making process should be the patient’s own preferences. If the patient is open to discontinuing medication, this can be explored. However, if the patient is not inclined to do so, it is important to respect that choice. As long as the patient wishes to continue the anticoagulant therapy, this option should remain available. (Participant 18, Medical oncologist)

### Solutions and recommendations

When asked about the support that could facilitate decision-making regarding anticoagulant therapy in the last phase of life, many healthcare professionals indicated that a guideline would be beneficial in decision-making and in supporting the shared decision-making process with the patient. While there was variation in opinions among healthcare professionals regarding the precise content of this guideline, nearly all emphasized that including numbers on the incidence of events should be an essential component. A smaller group of healthcare professionals indicated that they do not perceive a need for a guideline, as they are confident in their clinical decisions, grounded in their clinical reasoning and experience.

Regardless of the healthcare professionals’ opinion on the necessity of a guideline, most participants stated that the complexity of this multifactorial dilemma rendered the creation of a comprehensive guideline infeasible. Due to this complexity in decision-making, an individual patient cannot be entirely captured within a guideline.

Building on this, several healthcare professionals emphasized that future research should focus on studies stratified by indication for anticoagulant therapy and relevant comorbidities, to investigate the occurrence of thrombotic and bleeding events following the (dis)continuation of therapy in the last phase of life.

Given the complexity of this topic, the availability of more specific guidelines would be highly beneficial. Such guidelines could contain data on the incidence of ischemic stroke following the discontinuation of anticoagulant therapy. This would allow clinicians to move beyond intuition and base their decisions on evidence. Moreover, having access to these incidence rates would facilitate clearer and more informed discussions with patients. (Participant 10, Haematologist)

## Discussion

### Main findings

This in-depth interview study demonstrates that healthcare professionals regularly involved in care for patients in the last phase of life agree that anticoagulant therapy should be reconsidered in this phase. However, they experience decision-making regarding anticoagulant therapy in the last phase of life as difficult and multifactorial. The five most frequently cited difficulties were a lack of knowledge on the incidence of events after (dis)continuing anticoagulant therapy, the uncertain risk trade-off between two outcomes with major consequences, the variety of indications for anticoagulant therapy, the variety of relevant comorbidities and patient characteristics, and prognostic uncertainty. Uncertainty in decision-making results in reactive, rather than proactive, decision-making in response to triggers such as active bleeding, low performance score, recognition of the dying phase, and the patient’s preference.

### What this study adds?

This study highlights the complexity of decision-making regarding anticoagulant therapy in the last phase of life, as experienced by healthcare professionals. This is in line with findings from previous research.^13,26,27,30^ The variety of indications for anticoagulant therapy and corresponding medication strategies further demonstrates this complexity. For atrial fibrillation, guidelines recommend lifelong anticoagulant therapy for those with an elevated stroke risk determined by the CHA₂DS₂-VASc score.^21,22^ Recent studies have demonstrated that discontinuing anticoagulants prematurely in patients with atrial fibrillation is associated with an increased risk of ischemic stroke, even in those who have undergone successful catheter ablation.^33,34^

For prosthetic heart valves, guidelines recommend tailoring anticoagulant therapy based on the type of valve implanted. Mechanical valves necessitate lifelong vitamin K antagonists due to the persistent risk of valve thrombosis and systemic embolism. Bioprosthetic valves are generally treated with anticoagulant therapy for 3–6 months, after which therapy may be discontinued if no other indication exists.^35^ Interestingly, some studies have even questioned the benefit of anticoagulant therapy in this context.^36,37^

For venous thromboembolism, guidelines recommend at least 3 months of anticoagulant therapy. After this initial period, anticoagulation is typically discontinued in patients with a provoked event and continued in patients with an unprovoked event or persistent risk factor. Studies performed in the general population with a first unprovoked event have shown substantial recurrence rates after discontinuation of anticoagulant therapy after an initial period of therapy.^38–40^ In patients with active cancer, a persistent risk factor, discontinuation of therapy is associated with a high risk of recurrent venous thrombembolism.^41^ For those with unprovoked venous thromboembolism or persistent risk factors, extended or indefinite anticoagulation may therefore be considered. Recent studies have demonstrated that continuing reduced-dose anticoagulant therapy with direct oral anticoagulants, can effectively lower the rate of recurrent thromboembolic events while minimizing bleeding events.^42,43^

Most recommendations regarding (dis)continuing anticoagulant therapy are based on studies conducted in the general population or patients with cancer. The extrapolation of these recommendations to patients in the last phase of life can be questioned, given their higher risk of both thromboembolic and bleeding events compared to the general population.^16,44^ A cohort study among 18,000 vitamin K antagonist users with life-limiting diseases found similar rates of thromboembolic and bleeding events during and after anticoagulant use, suggesting that discontinuation of vitamin K antagonists may not significantly alter these risks.^7^

An important finding of this study is that many healthcare professionals perceived prognostic uncertainty as a limiting factor in decision-making regarding anticoagulant therapy. This applies in particular to illnesses other than advanced solid cancer, such as respiratory and heart failure, where illness trajectories are less predictable.^45^ However, prognostication in patients with advanced solid cancer is also becoming increasingly complex since the introduction of targeted therapy and immunotherapy, which can significantly influence illness trajectories.^46^

Another important finding that emerged from this study is that healthcare professionals consider patient preferences essential, highlighting the need to understand how patients perceive anticoagulant therapy in the last phase of life and how they wish to be involved in shared decision-making. This is therefore being studied as part of the OPTIMA project as well by interviewing patients and their relatives and also studies by the SERENITY consortium.^47^

### Limitations and strengths of the study

This in-depth interview study is the first study that provides profound insights into healthcare professionals’ considerations regarding decision-making about anticoagulant therapy in the last phase of life. Our results should be interpreted in the light of some limitations. First, selection bias may have been present, as participants were selected based on their willingness to participate, and might have been more aware of reconsidering medication use in the last phase of life. Second, the results may be influenced by social desirability bias because respondents may have given answers based on their views of what is most preferable or acceptable, potentially leading to underreporting of actual clinical behavior. Third, these results were obtained from healthcare professionals in a single European country. This may limit the generalizability of the findings, as practices regarding anticoagulant therapy in the last phase of life can vary across healthcare systems due to differences in drug availability and cost-related prescribing patterns.

## Conclusion

Decision-making regarding anticoagulant therapy in the last phase of life is experienced as difficult and multifactorial. This results, in practice, in reactive instead of proactive decision-making. Future research should focus on studies stratified by indication for anticoagulant therapy and relevant comorbidities to investigate the occurrence of thrombotic and bleeding events following the (dis)continuation of therapy in the last phase of life.

## Supplemental Material

sj-docx-1-pmj-10.1177_02692163251394875 – Supplemental material for Decision-making regarding anticoagulant therapy in the last phase of life: In-depth interview study with healthcare professionals (the OPTIMA trial) Supplemental material, sj-docx-1-pmj-10.1177_02692163251394875 for Decision-making regarding anticoagulant therapy in the last phase of life: In-depth interview study with healthcare professionals (the OPTIMA trial) by Marte A. M. van Hylckama Vlieg, Karlijn J. I. de Beukelaar, Romy H. L. Spreeuw, Marieke J. H. A. Kruip, P. Hugo M. van der Kuy, Agnes van der Heide, Carin C. D. van der Rijt and Eric C. T. Geijteman in Palliative Medicine
