# Supplementary material for: Decision-making regarding anticoagulant therapy in the last phase of life: In-depth interview study with healthcare professionals (the OPTIMA trial)
Source: Palliat Med. 2025 Nov 26;40(1):105–14. doi: 10.1177/02692163251394875 (PMC12779767; doi:10.1177/02692163251394875)
Supplement: sj-docx-1-pmj-10.1177_02692163251394875 – Supplemental material for Decision-making regarding anticoagulant therapy in the last phase of life: In-depth interview study with healthcare professionals (the OPTIMA trial) [file sj-docx-1-pmj-10.1177_02692163251394875.docx]

**Supplementary Material 1**

**Topic guide healthcare professionals**

Introduction

- Background and aim of the study
- Informed consent and audio recording
- Definitions of the last phase of life and anticoagulant therapy

Background information

- Profession and healthcare setting
- Experience with caring for patients in the last phase of life

Anticoagulant therapy in general

- Prescription of anticoagulant therapy and indication(s) for use
- Experiences with side effects of anticoagulant therapy
- Experiences with thrombotic events after discontinuation of anticoagulant therapy

Anticoagulant therapy in the last phase of life

- Perspectives on anticoagulant therapy during the last phase of life
- Experiences with side effects of anticoagulant therapy in the last phase of life
- Experiences with thrombotic events after discontinuation of anticoagulant therapy in the last phase of life

Decision-making regarding (dis)continuing anticoagulant therapy in the last phase of life

- General: reviewing and adjusting the medication list in the last phase of life
- General: identifying appropriate moments to critically review the medication list in the last phase of life
- Approach to anticoagulant use in the last phase of life
- Considerations around (dis)continuing anticoagulants in the last phase of life
- Influence of life expectancy on anticoagulant decision-making
  - 1 year
  - 6 months
  - 3 months
  - Final week(s)
- Influence of the type of anticoagulant on decision-making in the last phase of life
- Situations/ indications in which anticoagulants are continued in the last phase of life
- Situations/ indications in which anticoagulants are discontinued in the last phase of life
- Situations/indications that raise the most doubt about (dis)continuing anticoagulants in the last phase of life
- Previous experiences
- Involvement of the patient in anticoagulant decision-making
- Patient responses to the suggestion of (dis)continuing anticoagulant therapy

Existing knowledge and needs for further research

- Availability of clinical guidelines
- Use of clinical guidelines to support anticoagulant decision-making
- Perceived adequacy of guidelines in anticoagulant decision-making
- Additional knowledge or information needed to support anticoagulant decision-making
- Use of quantitative risk estimates (e.g., thrombotic/bleeding percentages) to support anticoagulant decision-making
- Suggestions for future research to support anticoagulant decision-making
